# Flow field characteristics and coal dust removal performance of an arc fan nozzle used for water spray

**DOI:** 10.1371/journal.pone.0203875

**Published:** 2018-09-20

**Authors:** Fangwei Han, Jian Liu

**Affiliations:** 1 College of Safety Science and Engineering, Liaoning Technical University, Huludao City, Liaoning Province, China; 2 Key Laboratory of Mine Thermodynamic Disasters and Control (Liaoning Technical University), Ministry of Education, Huludao City, Liaoning Province, China; Coastal Carolina University, UNITED STATES

## Abstract

Dust source that presents a ring shape is frequently observed in mining engineering. An arc fan nozzle used for water spray is designed to improve the dust removal efficiency. Based on a study of the spray field characteristics of an arc fan nozzle using volume of fluid (VOF) analysis, it is found that the section of arc fan flow gradually increases, and its mean width of the impact zone is 3.1 times that of the free jet zone. After leaving the guiding object, the central axis velocity of the arc fan flow rapidly increases and then gradually decreases. Based on dimensionless analysis, the calculation formula of the jet speed near the wall is achieved. The relationship between the section geometric features of arc fan flow and the structural parameters of the arc fan nozzle is analysed. A field test completed in the Fucun coal mine indicates that the dust removal efficiency obviously improved with the use of arc fan nozzles. Compared to the full cone nozzles used before, the average removal efficiency for total dust increased by 34%, and the average removal efficiency for respirable dust increased by 32%.

## Introduction

**Fig 1:**
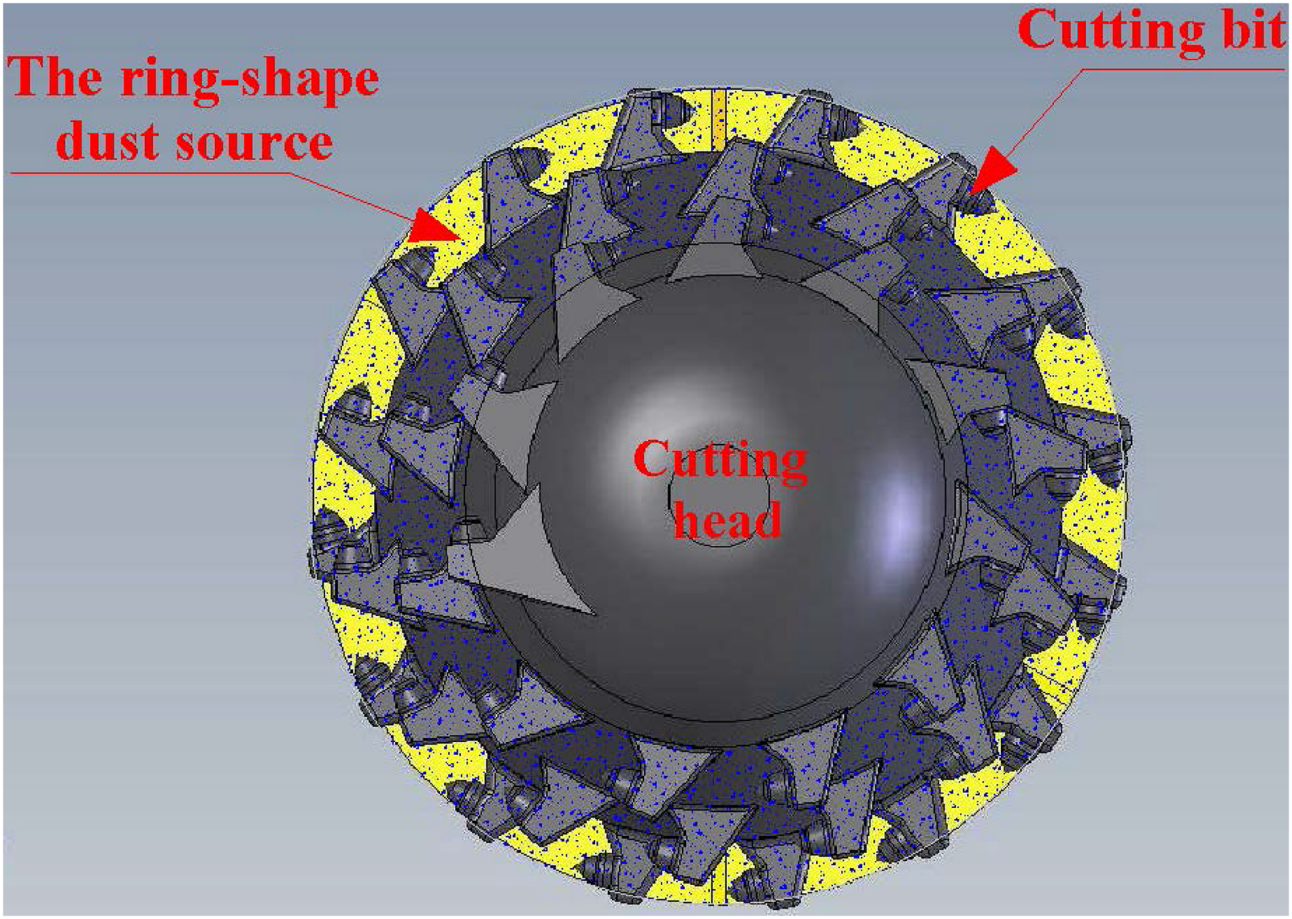
A ring-shaped dust source.

Dust source that presents a ring shape is frequently observed in mining process. The cutting operation of the vertical axis roadheader is a typical process in which a ring-shaped dust source will be generated, as shown in Fig 1. When the roadheader works, the cutting head will rotate and drive the cutting bits to rotate. Then the bits cut the coal wall. This will produce a ring of coal dust around the cutting head. Coal dust greatly pollutes the working environment and affects workers’ physical and mental health. Exposure to coal dust is a widespread hazard in a coal mine that can be expected to result in pneumoconiosis, an incurable malignant occupational disease [1, 2]. In addition, explosive coal dust could lead to explosion accidents with heavy casualties [3–7]. Of the existing dust removal methods, water spray, the earliest method of dust control in human society, has been extensively used because of the economic advantages and practicality [8–12]. There are two principles for dust removal with water spray: (a) the water wet material surfaces to prevent dust from becoming airborne, and (b) the spray knocks down dust that has been suspended in the air. When the dust particles are wetted by water, the weight of each particle will obviously increase. The possibility that the dust is taken away by the wind is greatly reduced. The sedimentation rate of the suspended dust is significantly improved.

Selecting the correct spray pattern is important in dust control practices. Spray pattern refers to the shape in which liquid droplets are distributed. Nozzles used for generating water spray can be categorized by the spray patterns they produce. The application nozzles in mining engineering are flat fan, hollow cone and full cone. Flat fan nozzles use a fan spray pattern with a narrow-strip impact area. They produce small-to-medium droplet sizes and are usually used in a narrow or enclosed space. Hollow cone nozzles have an annular impact area that can produce a medium-to-large diffusion angle. This type of nozzle is normally used to capture the dust in regions where particles have been scattered in the air. Full cone nozzles use a solid cone-shaped spray pattern with a round impact area. They produce medium-to-large droplet sizes. When the distance between the nozzle and the target is large, full cone nozzles usually have a better dust removal effect than the other nozzles [13].

Choosing the proper spray pattern is essential for effective dust control. However, an important problem of the treatment of a ring-shaped dust source generated by a longitudinal axis machine always exists. The best approach to suppressing the ring-shaped dust source is the use of an annular spray pattern. The spatial morphology of water spray, which is produced by flat fan nozzles and full cone nozzles, does not correspond to the ring-shaped dust source. The hollow cone nozzle can produce an annular impact area. However, the nozzle can only be placed on one side of the dust source, and the droplets will not be able to reach the other side of the rotating head. Thus, a set of nozzles are usually arranged around the rotating head. As a result, a group of hollow cone nozzles produce a set of small ring dusting areas that do not match the single large ring-shaped dust source produced by the rotating head.

To solve this problem, a higher spray pressure is usually adopted to ensure that as the droplet size decreases, the spray volume increases, thereby improving the efficiency of dust reduction [14, 15]. However, this method is energy intensive and water consuming. Moreover, a large number of droplets are not involved in the improvement of the working environment. As the droplet size decreases, the droplets become more susceptible to airflow and other factors. Therefore, it is not the best choice. The arc jet nozzle used for foam spray was designed to improve the dust removal efficiency of foam [16]. But this nozzle is not suitable for water spray. In this paper, an arc fan nozzle used for water spray is proposed. The flow field generated by this nozzle is represented as a three-dimensional arc fan in the space. The previous studies have reported that the flow field characteristics of nozzles have an important influence on the performance of dust removal [17–19]. So, we studied the flow field characteristics of this nozzle with numerical simulation. The dimensionless formula for calculating the jet speed near the wall is obtained. The relationship between the geometric characteristics of flow field and the critical structural sizes of the arc fan nozzle is analysed. The effect of dust removal is tested in the field.

## The structure of the arc fan nozzle used for water spray

**Fig 2:**
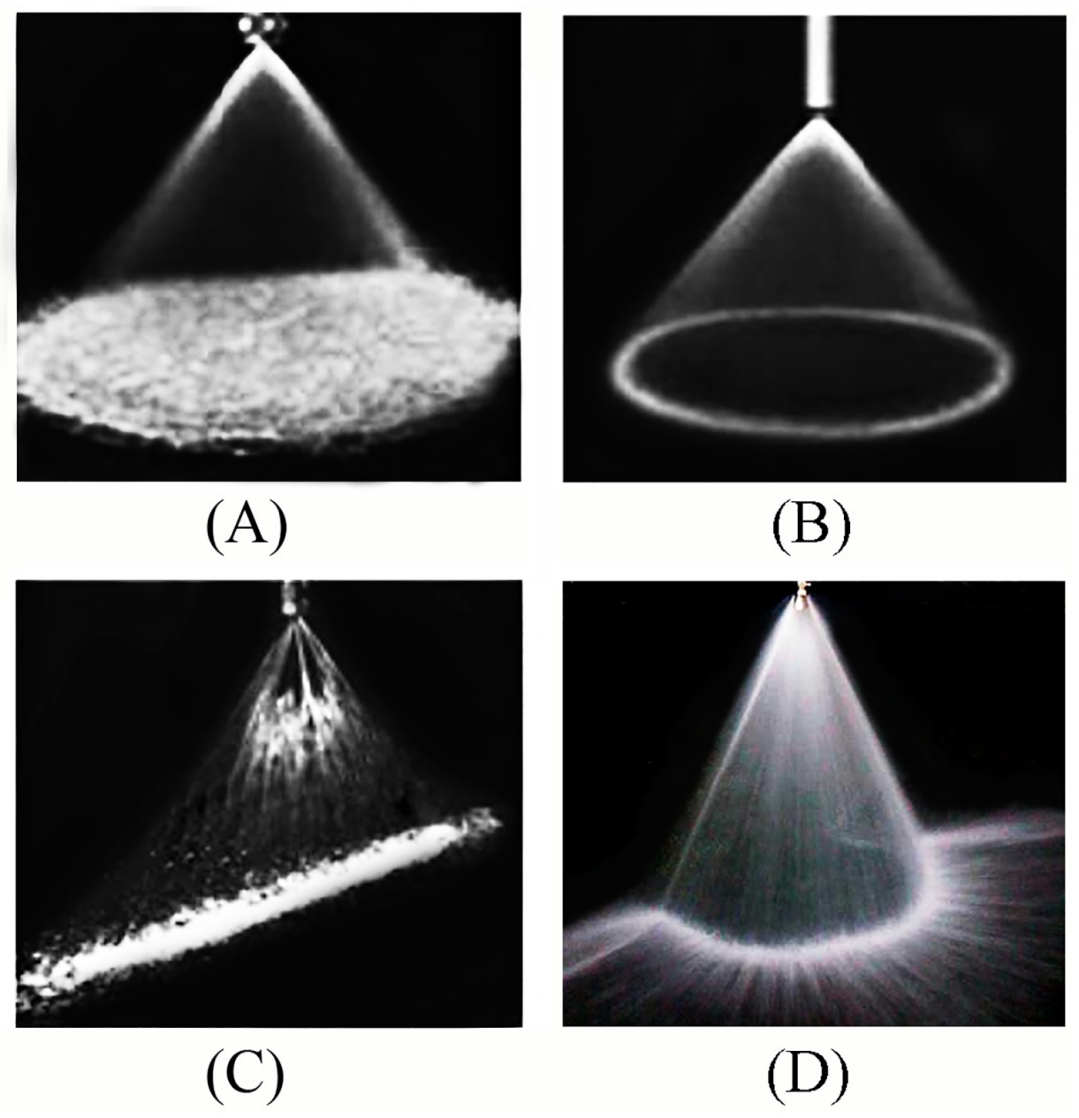
The water spray effect of four kinds of nozzles. (A) The water spray effect of full cone nozzle. (B) The water spray effect of hollow cone nozzle. (C) The water spray effect of flat fan nozzle. (D) The water spray effect of arc fan nozzle.

As mentioned, regardless of which conventional nozzle is used, the spray pattern is not consistent with the geometric shape of the ring-shaped dust source. For this reason, a water spray nozzle that can produce an arc-shaped spray pattern is developed to accurately and efficiently control a ring-shaped dust source. The water spray effect of full cone nozzle, hollow nozzle, flat fan nozzle and arc fan nozzle is respectively shown in Fig 2.

**Fig 3:**
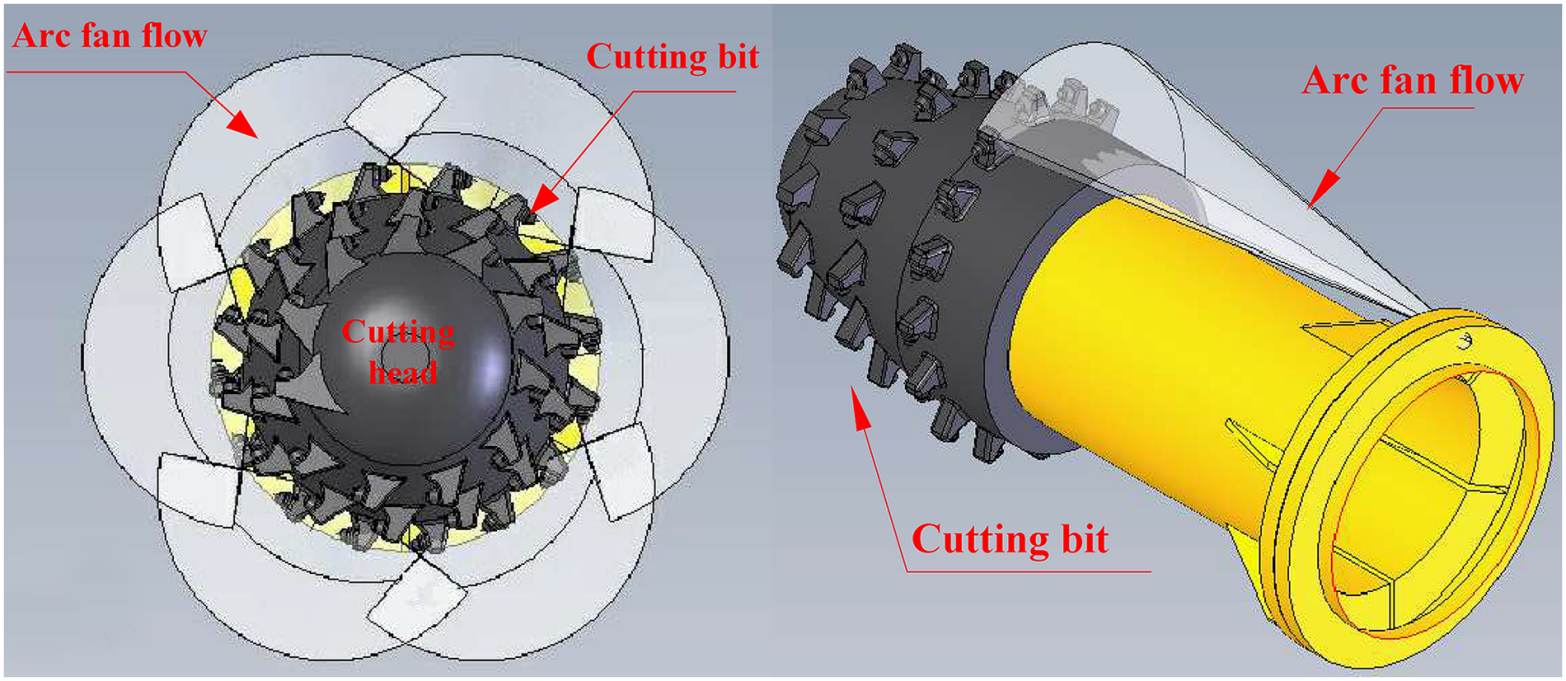
The ring-shaped dust source is suppressed by a group of arc fan flows.

The ring-shaped dust source is divided into several segments of arc dust sources. Each arc dust source is suppressed by an arc fan flow, which is generated by an arc fan nozzle, as shown in Fig 3. In this manner, each arc fan flow can correspond highly to one arc dust source.

**Fig 4:**
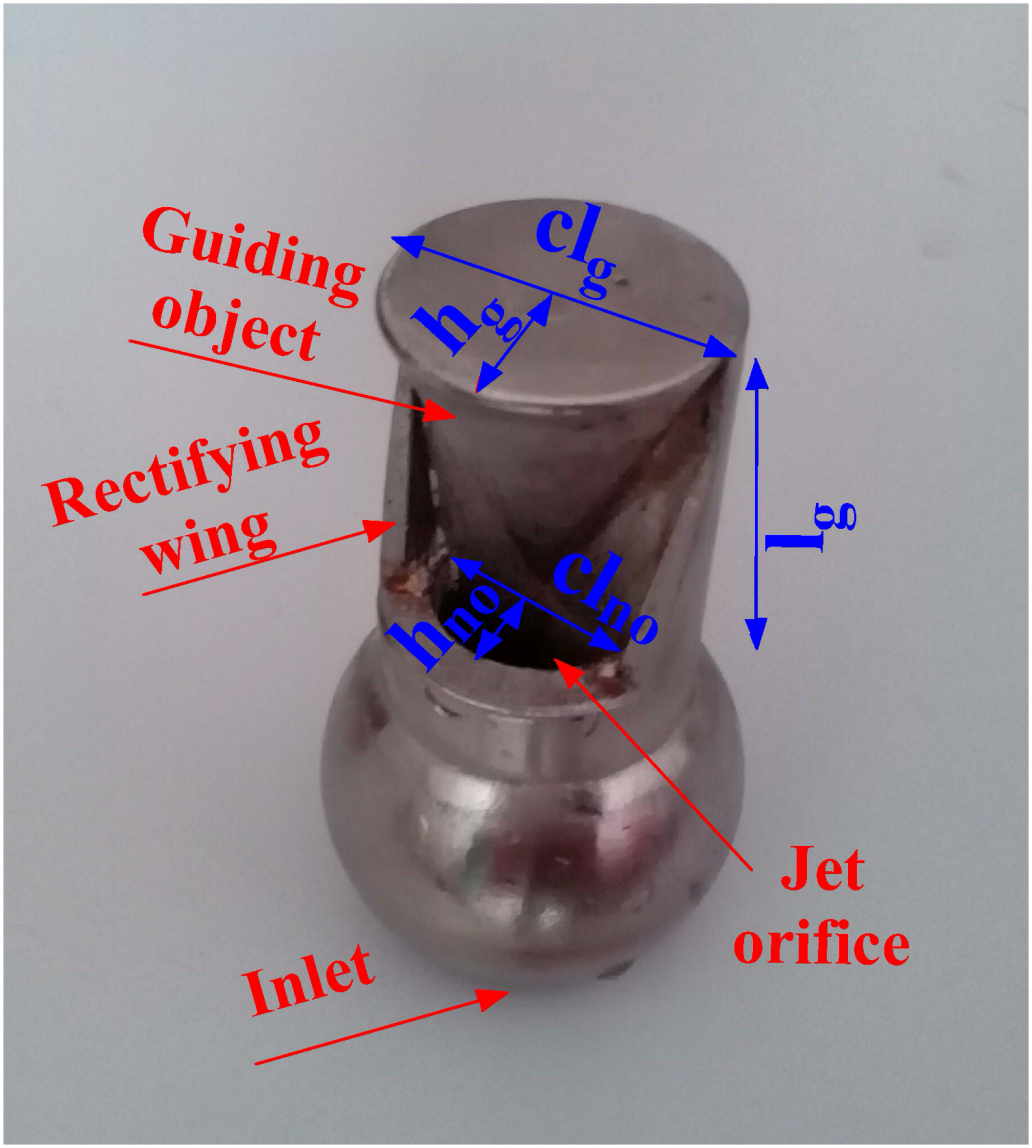
The structure of the arc fan nozzle.

The structure of the arc fan nozzle is illustrated in Fig 4. It is mainly composed of an inlet, a jet orifice, a pair of rectifying wings and a guiding object. The spray characteristics of the nozzle are specifically influenced by the geometry of the spray holes. Thus, the arc fan nozzle used for water spray is designed with an arched jet orifice. The fundamental parameters of the nozzle are the chord length of the nozzle orifice (*cl*_*no*_), the height of the nozzle orifice (*h*_*no*_), the length of the guiding object (*l*_*g*_), the chord length of the guiding object (*cl*_*g*_) and the height of the guiding object (*h*_*g*_). The chord length of the nozzle orifice (*cl*_*no*_) and the height of the nozzle orifice (*h*_*no*_) determine the area of the jet orifice, which affects the mist flux. To meet the demand of field conditions in a coal mine, *cl*_*no*_ was set as 10 mm, and *h*_*no*_ was set as 5 mm. The length of the guiding object (*l*_*g*_), the chord length of the guiding object (*cl*_*g*_) and the height of the guiding object (*h*_*g*_) are the fundamental dimensions to determine the geometrical characteristics of the arc fan flow. They can affect the coverage performance of the water. When *l*_*g*_ is greater than 35 mm, the nozzle is easily impacted by foreign objects and is likely to be damaged. When *l*_*g*_ is less than 25 mm, forming the ideal arc spray is often difficult because the guide distance is too short. Therefore, *l*_*g*_ is set to 30 mm. *cl*_*g*_ and *h*_*g*_ must be set according to the situation of the dedusting site.

**Fig 5:**
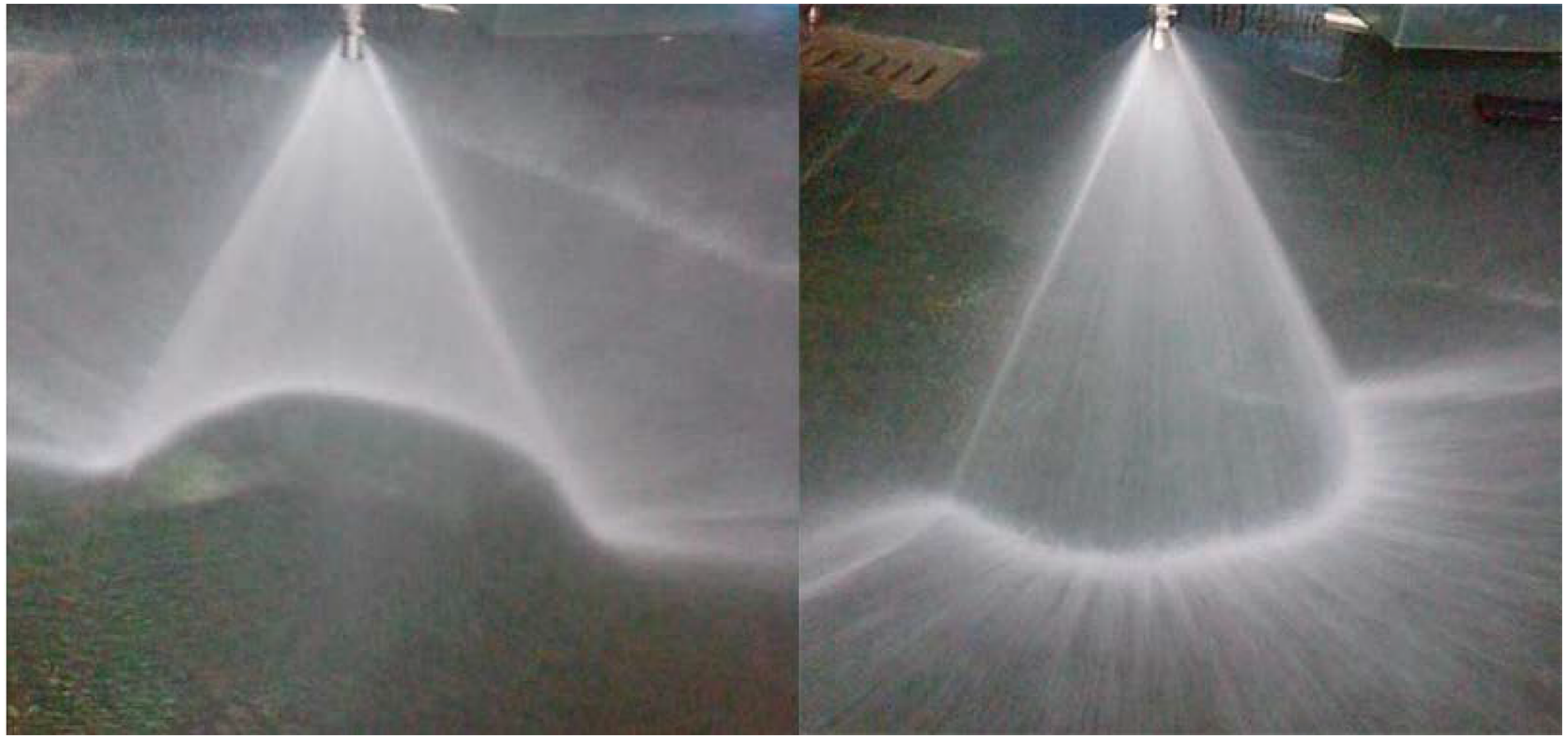
The water spraying effect of the arc fan nozzle.

The water ejected from the arched jet orifice impinges on the guiding object to form an arc-like flow. The guiding object is located outside the nozzle. It is designed to control the spray pattern. The section of the guiding object is an arc, which is similar to the section of the nozzle orifice. This approach is conducive to forming the stable arc fan flow. Fig 5 shows the water spraying effect.

## Numerical analysis of the flow field characteristics

### Simulation methods and parameter setting

The main goal of this numerical research is to understand the flow formation process and resolve the spatial characteristics of the flow field. The key characteristic dimension of flow field is critical to understand the dedusting performance of arc fan nozzle. It helps in achieving the best design and performance of the nozzle. So, prediction of the phase interface between the water and the air is essential to the successful computation of the arc fan flow. By coupling the VOF method and the standard k~ε turbulence model, the flow field of the arc fan nozzle is simulated. Unlike many other methods developed to flow with the interface, the VOF method can model the interface and the coalescence without special treatment. In the VOF model, a single set of momentum equations is solved for both fluids. By defining and using the VOF function to reconstruct the moving interface, the accurate position of the interface can be obtained [20, 21].

In the numerical simulation of the flow field of the arc fan nozzle, the air is the first phase, the water is the second phase, and the flow interface is tracked by the change of the VOF function with time. The basic governing equations are as follows.

① Volume fraction equation

$$\alpha_{l}+\alpha_{g}=1$$ (1)

where *α*_*l*_ and *α*_*g*_ are the volume fractions of water and air, respectively.

The calculation formula of *α*_*l*_ is shown below.

$$\frac{\partial \alpha_{l}}{\partial t}+\upsilon ∙{\nabla \alpha }_{l}=0$$ (2)

② Continuity equation

$$\frac{\partial \rho }{\partial t}+\nabla ∙(\upsilon ∙\rho )=0$$ (3)

where υ is the mass-averaged velocity, *ρ* is the mixture density, and t is time.

③ Momentum equation

The momentum equation is determined by the volume fractions of all phases through the properties *ρ* and *μ*.

$$\frac{\partial }{\partial t}(\rho u_{i})+\frac{\partial \rho }{\partial x_{j}}(\rho u_{i}u_{j})=-\frac{\partial p}{\partial x_{i}}+\frac{\partial \tau_{ij}}{\partial x_{j}}+\rho g_{i}$$ (4)

where *u*_*i*_ is the velocity component, *x*_*i*_ and *x*_*j*_ are coordinate components, *p* is the static pressure, *τ*_*ij*_ is the stress tensor, and *g*_*i*_ is the gravitational body force along the *i* direction.

④ Energy equation

$$\frac{\partial }{\partial t}(\rho E)+\frac{\partial }{\partial x_{i}}[u_{i}(\rho E+p)]=\frac{\partial }{\partial x_{i}}(k_{eff}\frac{\partial T}{\partial x_{i}})$$ (5)

where E is energy, T is temperature, and *k*_*eff*_ is the effective thermal conductivity.

⑤ Turbulence model

The standard k~ε turbulence model is applied to all phases:

$$\frac{\partial }{\partial t}(\rho k)+\frac{\partial }{\partial x_{i}}(\rho ku_{i})=\frac{\partial }{\partial x_{j}}[(\mu +\frac{\mu_{t}}{\sigma_{k}})\frac{\partial k}{\partial x_{j}}]+G_{k}+G_{b}-\rho \varepsilon -Y_{M}$$ (6)

$$\frac{\partial }{\partial t}(\rho \varepsilon )+\frac{\partial }{\partial x_{i}}(\rho \varepsilon u_{i})=\frac{\partial }{\partial x_{j}}[(\mu +\frac{\mu_{t}}{\sigma_{\varepsilon }})\frac{\partial \varepsilon }{\partial x_{j}}]+G_{1\varepsilon }\frac{\varepsilon }{k}(G_{k}+G_{3\varepsilon }G_{b})-G_{2\varepsilon }\rho \frac{\varepsilon^{2}}{k}$$ (7)

The term *G*_*k*_ indicates the generation of turbulence kinetic energy corresponding to the mean velocity gradients. The term *G*_*b*_ represents the generation of turbulence kinetic energy corresponding to the buoyancy. The term *Y*_*M*_ denotes the contribution of the fluctuating dilatation in compressible turbulence to the overall dissipation rate.

The turbulent viscosity *μ*_*t*_ is calculated by formula (8):

$$\mu_{t}=\rho C_{\mu }\frac{k^{2}}{\varepsilon }$$ (8)

In these equations, the values of constants *G*_1*ε*_, *G*_2*ε*_, *G*_3*ε*_, and *C*_*μ*_ are 1.44, 1.92, 0.09, and 0.90, respectively. The values of the turbulent Prandtl numbers *σ*_*k*_ and *σ*_*ε*_ are 1.0 and 1.3, respectively.

In addition, the SIMPLEC algorithm is used to solve the flow field. The arc fan nozzle outlet velocity is set to 10 m/s. The target distance is set to 1 m. The chord length of the nozzle orifice and the height of the nozzle orifice are 10 mm and 5 mm, respectively. The length of the guiding object is 30 mm. The chord length of the guiding object and the height of guiding object are 20 mm and 10 mm, respectively.

### Results and discussion

#### (1) Flow velocity of the mixed phase and water volume fraction

**Fig 6:**
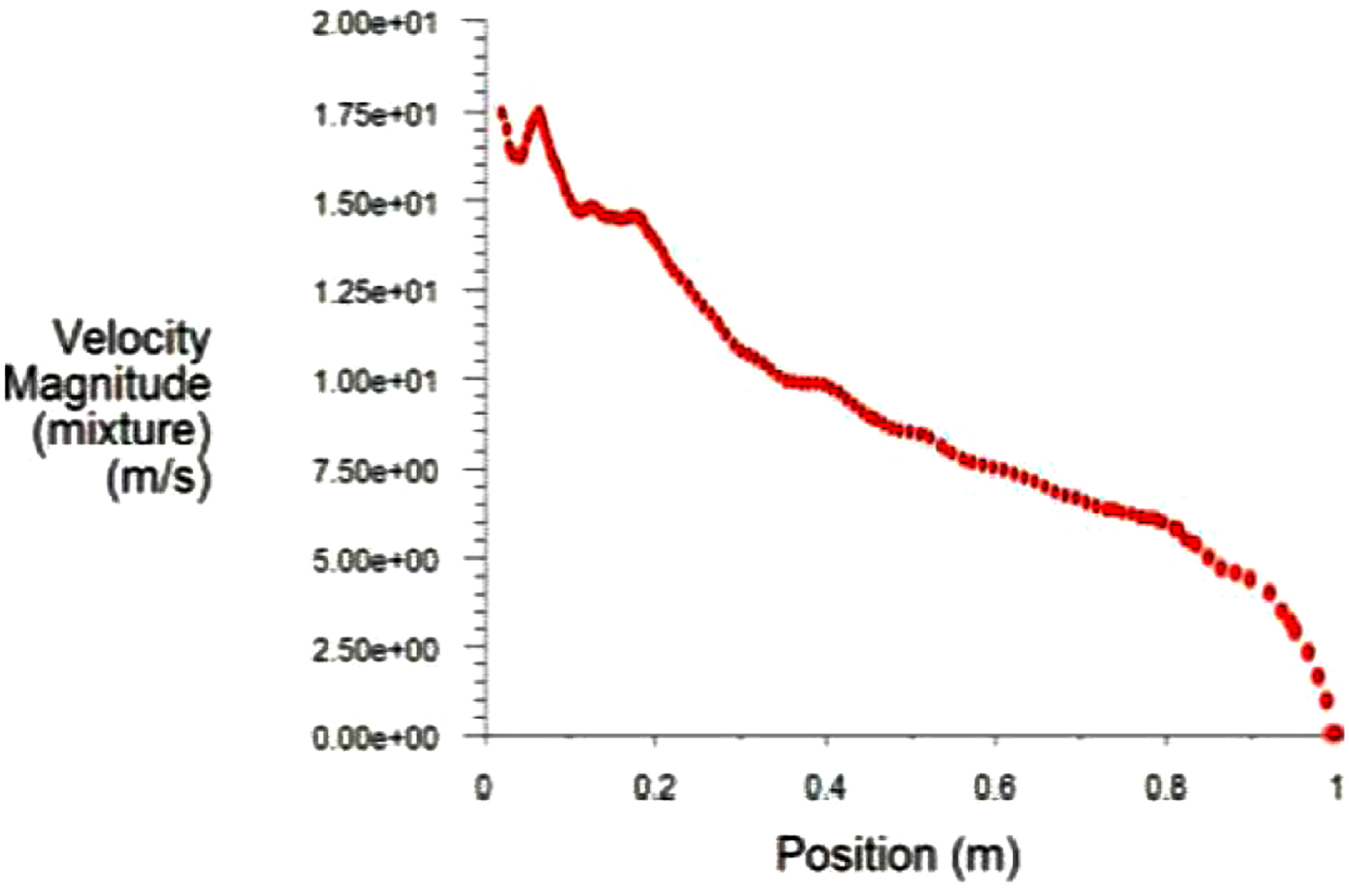
The flow velocity of mixed phase in monitoring line.

As the water transports gradually outward, water and gas are gradually mixed. In the practice of dust removal, both water and gas will interact with dust particles. It is meaningful to study the velocity of the mixed phase. Take the central axis of arc fan flow as the monitoring line. The velocity of the mixed phase in the monitoring line can be obtained, as shown in Fig 6.

After leaving the guiding object, the flow velocity is increased in seconds. With the interaction of water and gas, the flow velocity decreases gradually. Within 0.1 m from the wall, the flow velocity suddenly decreases. This zone is the impact zone. The numerical simulation results of the flow velocity in the impact zone are derived, and the non-dimensional analysis is conducted. Next, the following formula is obtained.

$$\frac{u_{m}}{u_{0}}\sqrt{\frac{L}{2d}}=5.78\sqrt{1-\frac{x}{L}}$$ (9)

where *x* indicates the distance from the spray hole, *L* is the distance between the nozzle orifice and the wall, *u*_0_ is the exit velocity at the nozzle orifice, *d* is the equivalent diameter of the nozzle orifice, and *u*_*m*_ is the flow velocity near the wall. When *L*, *u*_0_, and *d* are known, the flow velocity near the wall can be calculated by using formula (9).

**Fig 7:**
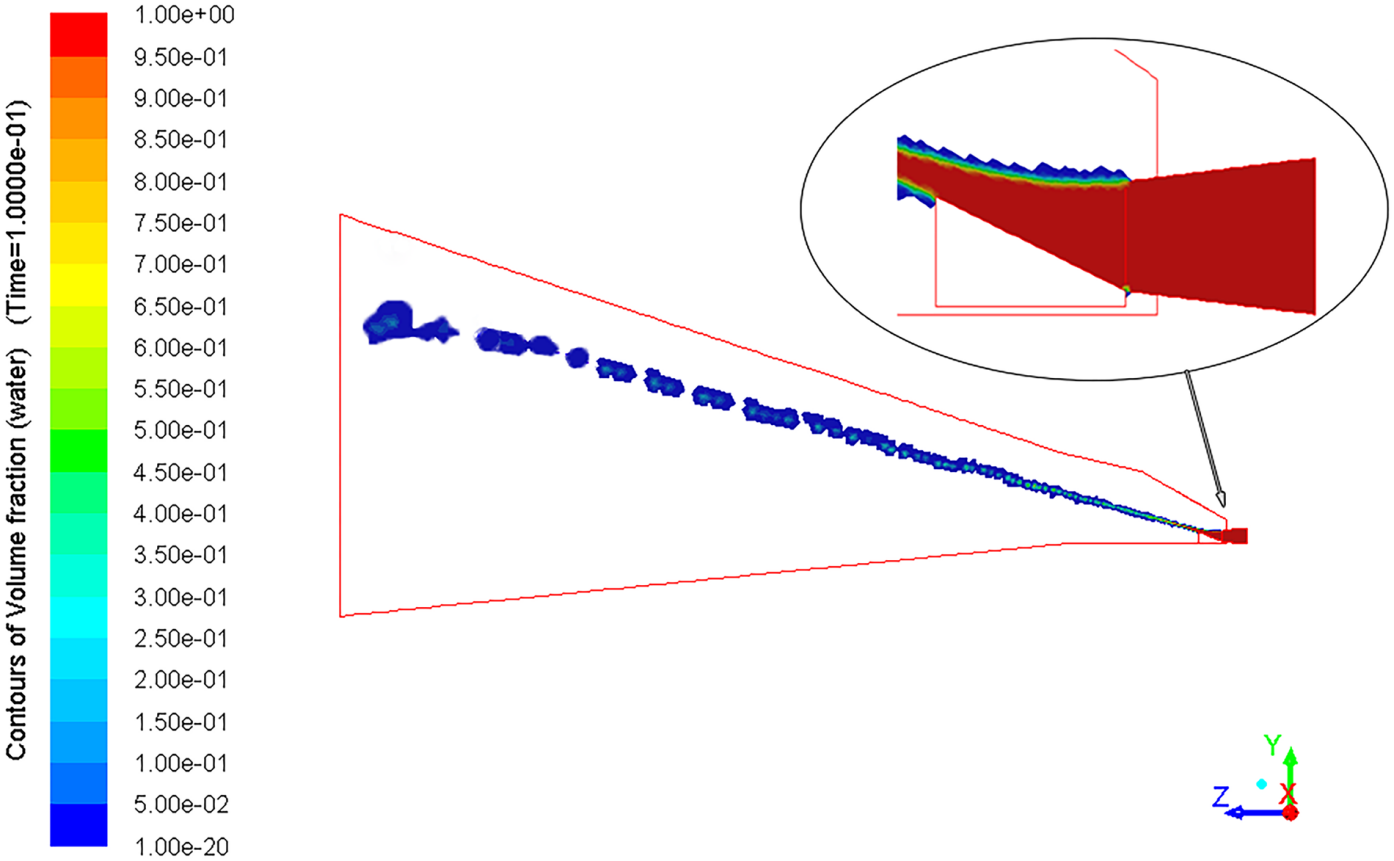
Contours of water volume fraction at the axial section (x = 0 m).

Fig 7 shows the change of water volume fraction at the axial section (x = 0 m). It can be seen that at the initial stage of water jet formation, the water volume fraction is 1. With the gradual development of the jet, water and gas mixed gradually. The water volume fraction gradually decreases. At the location of the liquid film rupture, the water volume fraction is 0. The droplets are produced when the water film is gradually broken.

#### (2) The cross-sectional area of the arc fan flow

**Fig 8:**
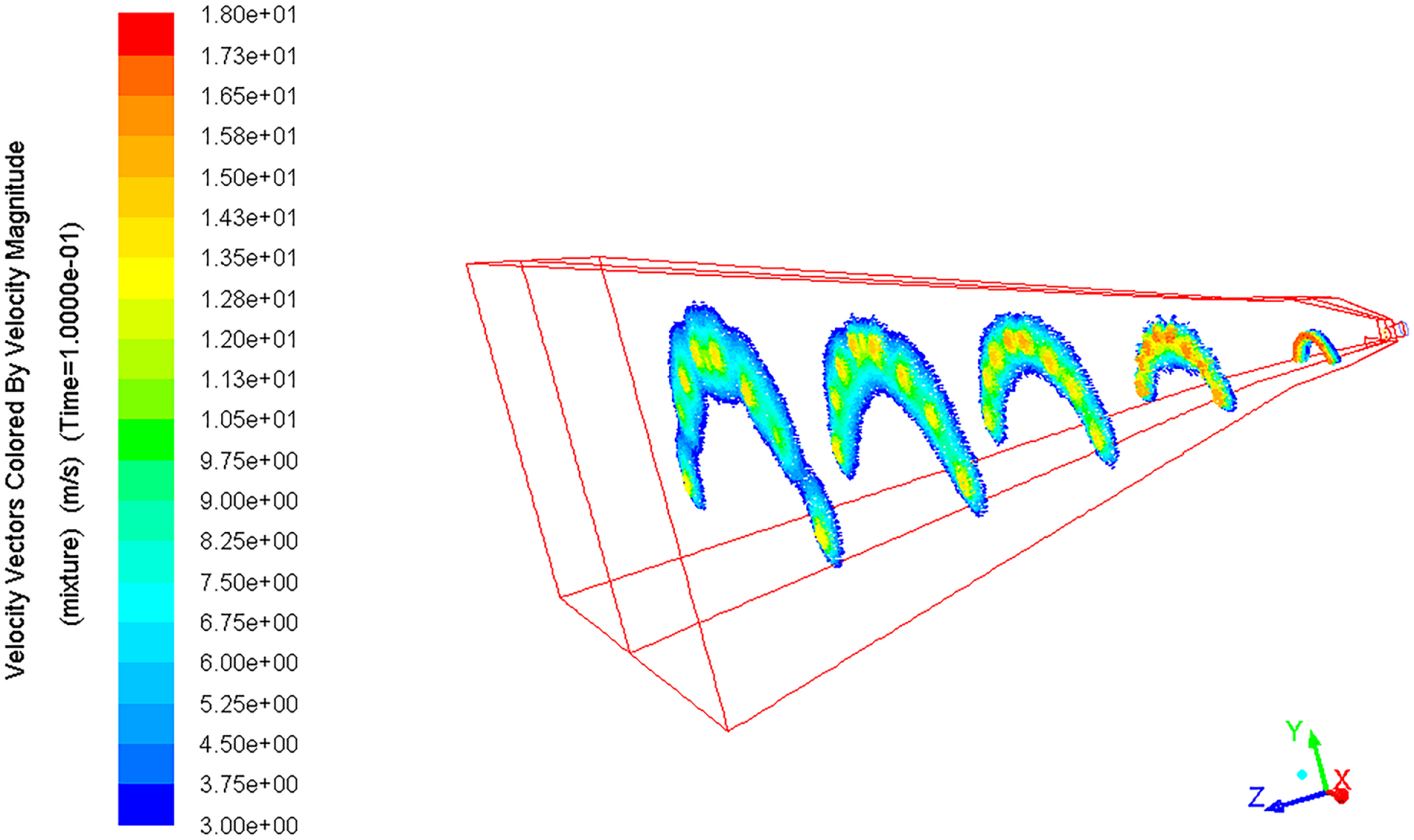
Velocity distribution of mixed phase at multiple cross sections.

**Fig 9:**
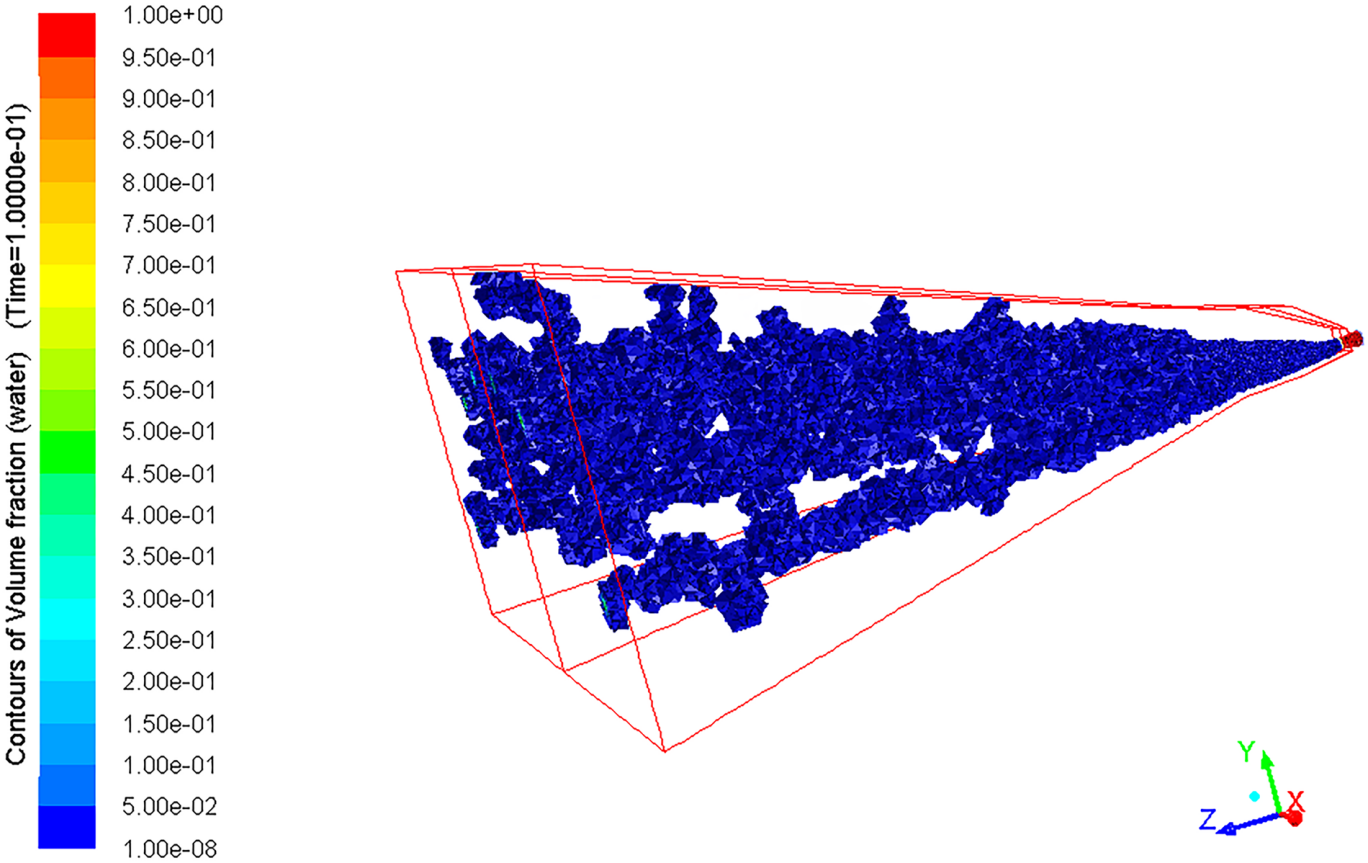
Water distribution in the spray space.

As shown in Figs 8 and 9, the flow generated by the arc fan nozzle is represented as a three-dimensional arc fan in the space. Moreover, the cross section of the arc fan is arched.

As the water flows away from the guiding object, it experiences two other stages: the free jet zone and the impact zone. In the free jet zone, the cross-sectional area of the arc fan flow increases gradually. It is mainly because of the interaction between the water flow and the surrounding air. In addition, the surface tension of the water also plays a role in the breakup of the water flow. The curvature radius of the liquid surface is changing with the change of the static pressure. The uneven distribution of pressure causes the liquid to break. The water is gradually dispersed and the boundary of the jet is expanding [22].

In the impact zone, the arc fan flow hits the wall, causing the movement direction to change. After impacting the frontal wall, under the driving force of the pressure gradient, the water will spread out of the initial impact region rapidly. This significantly increases the geometric dimension of the flow in the impact zone. The average cross section width of the impact zone is 3.1 times that of the free jet zone. With the increase of the cross-sectional area, the range of dust control is expanded.

**Fig 10:**
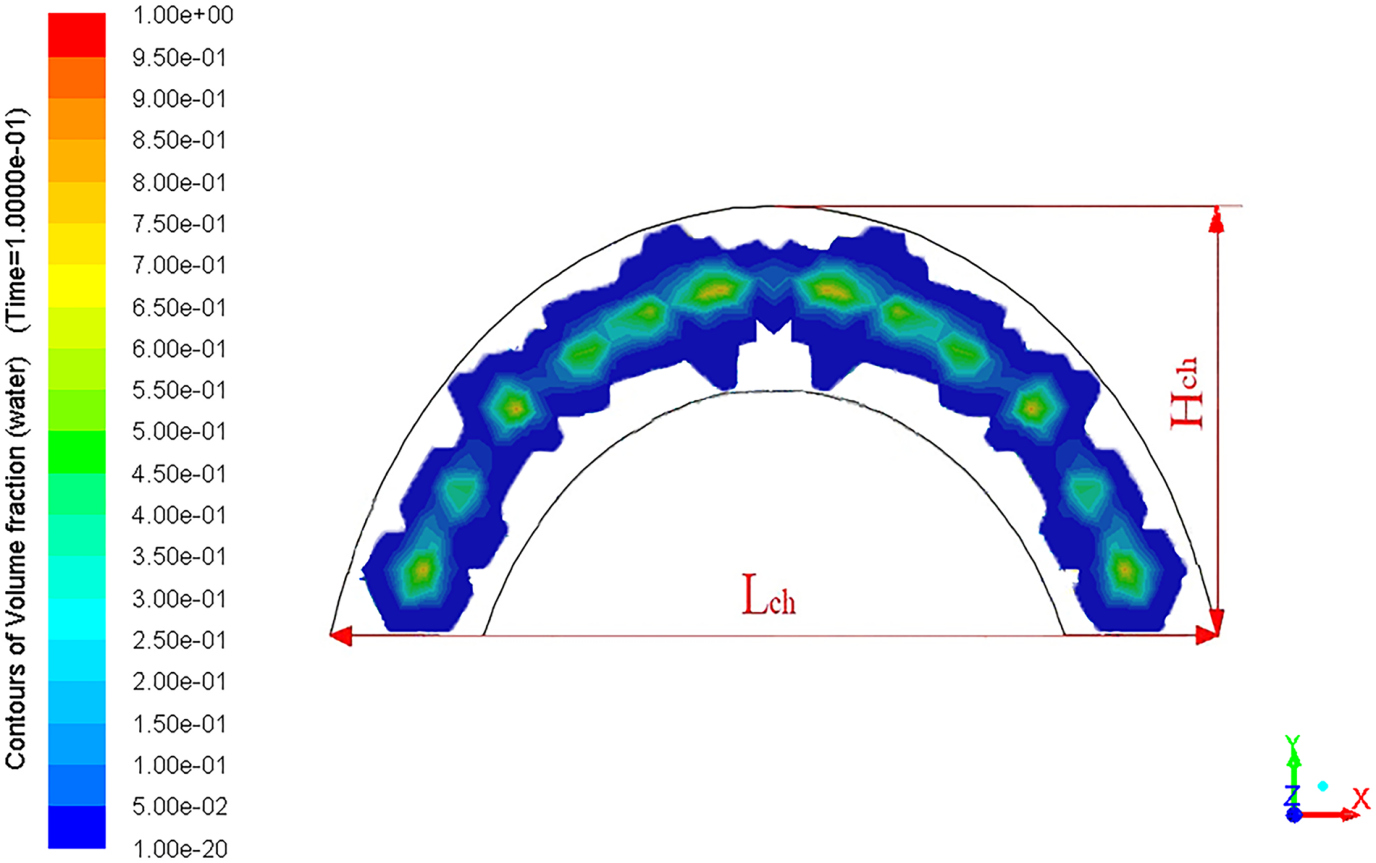
The sectional geometric features of the arc fan flow.

As shown in Fig 10, *L*_*ch*_ is defined to describe the maximum chord length of the cross section. *H*_*ch*_ is defined to describe the maximum perpendicular distance from the vertex of the arc to *L*_*ch*_. *L*_*ch*_ and *H*_*ch*_ jointly reflect the maximum coverage area of the arc fan flow. Based on the spatial geometry, the functional relations between sections of the arc fan flow and the nozzle structural parameters are deduced, as expressed in formulas (10) and (11).

$${cl}_{g}=\frac{L_{ch}\times l_{g}}{\alpha \times L}$$ (10)

$$h_{g}=\frac{H_{ch}\times l_{g}}{\beta \times L}$$ (11)

In the practical situations, the migration trajectory of water can be influenced by many factors, such as air resistance and wall impingement. It is difficult for the water to flow completely according to the spatial geometry. Thus, two factors, *α* and *β*, are used to make the necessary corrections.

## Experimental investigation of the correction factors

### Experimental systems and experimental procedures

**Fig 11:**
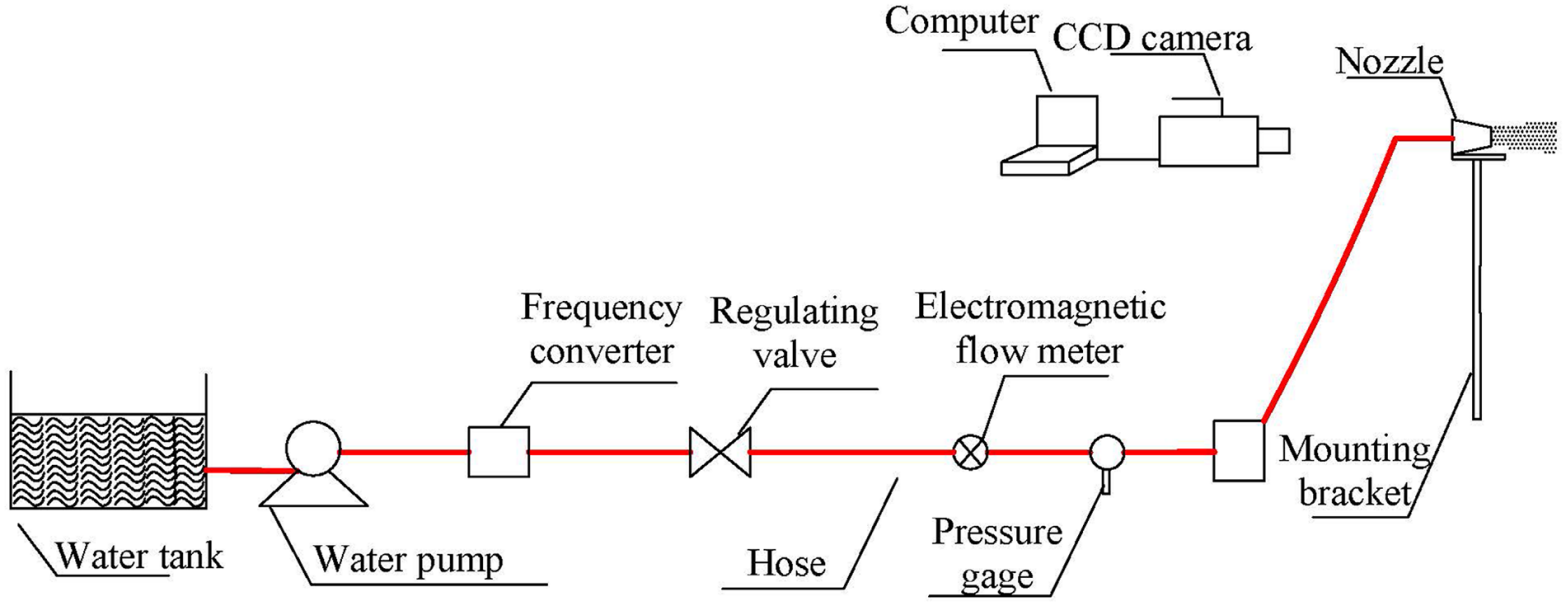
The experimental arrangement.

Laboratory experiments were conducted to obtain *α* and *β*. The experimental arrangement is illustrated schematically in Fig 11. The spray system consists of a water tank, a water pump, a frequency converter, a regulating valve, a mounting bracket and a nozzle. The test instruments include a pressure gage, an electromagnetic flow meter, a CCD camera and a computer.

The sprayed water is pumped out from the water tank. The sprayed mass flow rate is controlled by adjusting the frequency converter and the regulating valve located at the discharge line of the water pump. Flow rate is measured by the electromagnetic flow meter. Pressure is monitored through the pressure gauge. Water is sprayed into the room temperature and atmospheric pressure environment through the arc fan nozzle. The arc fan nozzle was mounted on a bracket. The design dimensions of the nozzles applied in experiments are shown in Table 1. The spray process was recorded by the CCD camera at 5ms intervals (200 fps). The resolution of captured images was 1028 × 800 pixels, with which *L*_*ch*_ and *H*_*ch*_ can be measured.

**Table 1:** The design dimensions of the arc fan nozzles applied in the experiments.

	*cl*_*no*_/mm	*h*_*no*_/mm	*l*_*g*_/mm	*cl*_*g*_/mm	*h*_*g*_/mm
**#1**	10	5	30	16	8
**#2**	10	5	30	17	8.5
**#3**	10	5	30	18	9
**#4**	10	5	30	19	9.5
**#5**	10	5	30	20	10
**#6**	10	5	30	21	10.5
**#7**	10	5	30	22	11
**#8**	10	5	30	23	11.5
**#9**	10	5	30	24	12
**#10**	10	5	30	25	12.5

The experimental procedures in this part are as follows:

(1) Assemble the experimental systems according to Fig 11. Install nozzle #1 on the bracket. The distance between the nozzle orifice and the wall is set to 1 m.
(2) Start the water pump. Adjust the water flow by using a frequency converter. Maintain the rated water pressure at 1 MPa.
(3) Trigger the CCD camera to begin recording.
(4) Adjust the water supply to 2 MPa and to 3 MPa.
(5) Stop the camera, and change the nozzle to #2, #3, #4, etc. Then, repeat steps (2), (3) and (4).
(6) Finally, analyse the time-series images captured via the CCD camera. Record the values of *L*_*ch*_ and *H*_*ch*_ obtained under different conditions.

### Results and discussion

Through the experimental tests, *L*_*ch*_ and *H*_*ch*_ under different conditions were obtained. Other relevant parameters, *l*_*g*_ and *L*, were determined at the same time. The correction factors *α* and *β* can be calculated by using formulas (10) and (11). The testing data and calculated results are presented in Table 2.

**Table 2:** The testing data and calculated results.

	Water pressure of 1 MPa.				Water pressure of 2 MPa.				Water pressure of 3 MPa.			
	*L*_*ch*_/mm	*H*_*ch*_/mm	*α*	*β*	*L*_*ch*_/mm	*H*_*ch*_/mm	*α*	*β*	*L*_*ch*_/mm	*H*_*ch*_/mm	*α*	*β*
**#1**	405	125	0.76	0.47	430	130	0.81	0.49	475	150	0.89	0.56
**#2**	416	138	0.73	0.49	447	145	0.79	0.51	483	168	0.85	0.59
**#3**	435	149	0.73	0.50	520	152	0.87	0.51	535	172	0.89	0.57
**#4**	458	161	0.72	0.51	525	175	0.83	0.55	552	195	0.87	0.62
**#5**	485	180	0.73	0.54	536	210	0.80	0.63	585	208	0.88	0.62
**#6**	508	195	0.73	0.56	570	235	0.81	0.67	602	245	0.86	0.70
**#7**	555	210	0.76	0.57	628	243	0.86	0.66	665	265	0.91	0.72
**#8**	590	221	0.77	0.58	670	262	0.87	0.68	705	279	0.92	0.73
**#9**	615	238	0.77	0.60	710	280	0.89	0.70	735	300	0.92	0.75
**#10**	648	245	0.78	0.59	718	295	0.86	0.71	752	315	0.90	0.76

It can be found that *L*_*ch*_ and *H*_*ch*_ maintain the same change tendency with *cl*_*g*_ and *h*_*g*_. Both *L*_*ch*_ and *H*_*ch*_ were found to increase gradually with the water pressure. The correction factor *α* was found to range from 0.72 to 0.92, with an average value of 0.82. *β* was found to range from 0.47 to 0.76, with an average value of 0.60. The average values were chosen as the target values. Thus, formulas (12) and (13) can be obtained by getting the average values into formulas (10) and (11).

$${cl}_{g}=\frac{L_{ch}\times l_{g}}{0.82L}$$ (12)

$$h_{g}=\frac{H_{ch}\times l_{g}}{0.6L}$$ (13)

## Field experiment

As mentioned above, the arc fan nozzle used for water spray realized an arc spray pattern. A field test was performed to investigate its dust control performance. The field test was performed in an underground roadway of the Fucun coal mine (located at 34.854°N and 117.059°E), belonging to Shandong Energy Group in China. This coal mine gave us the permission to conduct the study. The cross section of the roadway was a rectangle of 13.3 m^2^ (3.8 m wide and 3.5 m high). A vertical axis roadheader was driven to advance in the roadway. It cut 63.84 m^3^ of coal at the face every day.

The roadheader uses 6 full cone nozzles formerly used for dust control. During the comparative experiment, the water supply was kept constant. The number of the arc fan nozzles and the installation positions thereof were similar to those of the formerly used full cone nozzles.

As the maximum cutting diameter was 1050 mm, the required *L*_*ch*_ was 525 mm, and the required *H*_*ch*_ was 70.4 mm. According to the nozzle installation positions, the measured distance of the spray target (L) was 1000 mm. According to formulas (12) and (13), the chord length of the guiding object (*cl*_*g*_) and the height of the guiding object (*h*_*g*_) were 19.2 mm and 3.5 mm, respectively. The actual coverage performance of this arc fan nozzle was verified in the laboratory. The test results showed that the maximum coverage chord length *L*_*ch*_ satisfied the condition, whereas the maximum arch height *H*_*ch*_ failed to reach the expected value. To achieve the expected performance, nozzles with different *h*_*g*_ values were examined. The nozzle with *cl*_*g*_ = 19.2 mm and *h*_*g*_ = 9 mm was determined to be the final choice.

To obtain dust concentrations under different conditions, a sampling point was positioned at the driver position when the roadheader was operational. The choice of sampling site conforms to the standards of safety production in People’s Republic of China [23]. The testing point was 1.7 m above the floor of this roadway. This value is a typical height of a worker’s breathing zone in the Fucun coal mine. The total dust concentrations (TDCs) and respirable dust concentrations (RDCs) were measured by a direct reading dust detector (CC-20, Xuzhou Jiangmei Technology Co. Ltd.). The instrument has the advantages of automatic timed sampling, convenience and reliability. The sampling flow in the test was 20 L/min. The sampling time for each test was 60 s.

In the location of the roadheader drivers, TDCs and RDCs were measured for three different conditions, namely, a) no treatment measures; b) use of the former full cone nozzles; and c) use of the arc fan nozzles. To reduce the test error, five groups of dust concentrations were tested under each condition. Next, the average values were calculated by using these five groups of measured data.

The corresponding dust removal efficiency can be obtained by using formula (14).

$$\eta =\frac{C_{1}-C_{2}}{C_{1}}\times 100\%$$ (14)

In formula (14), *η*(%) is the dust suppression efficiency, *C*_1_(mg/m^3^) is the dust concentration before applying dust control technology, and *C*_2_(mg/m^3^) is the dust concentration after applying dust control technology [24].

The results are shown in Table 3.

**Table 3:** Experimental and computational results under various conditions.

	No spraying		Spray by full cone nozzles		Spray by arc fan nozzles	
	TDC	RDC	TDC	RDC	TDC	RDC
**Measured data (mg/m³)**	815.6	333.6	296.6	156.5	110.3	65.2
	806.5	367.4	250.3	115.6	79.7	40.6
	810.4	344.6	263.2	105.3	82.5	43.2
	806.2	377.8	291.8	141.3	109.9	53.2
	802.8	360.1	285.6	97.8	98.6	38.8
**Average value (mg/m³)**	808.3	356.7	277.5	123.3	96.2	48.2
**The average dedusting efficiency(%)**			65.7	65.4	88.1	86.5

Experimental and computational results show that the average dedusting efficiencies for the total dust and the respirable dust using the arc fan nozzle were 88.1% and 86.5%, respectively. Compared to the full cone nozzles used before, the average removal efficiency for the total dust increased by 34%, and the average removal efficiency for the respirable dust increased by 32%. The results show that, under the unchanged water supply, the utilization efficiency of water sprayed by the arc fan nozzle is improved obviously, and the effect of dust removal is improved remarkably. These results of the field experiment prove that this arc fan nozzle is very appropriate for producing water spray to control a ring-shaped dust source.

## Conclusions

The flow field characteristics and the coal dust removal performance of an arc fan nozzle were studied via numerical analysis and experimental investigation. The conclusions of this study are as follows:

1. To achieve efficient and accurate dust removal, the ring-shaped dust source is divided into several segments of arc dust sources. Each arc dust source is suppressed by an arc water flow, which is generated by an arc fan nozzle.
2. The VOF method is used to simulate the flow field of the arc fan nozzle; the results show that the cross-sectional area of the arc fan flow gradually increases. Its mean width of the impact zone is 3.1 times that of the free jet zone. After leaving the guiding object, the central axis velocity of the arc fan flow rapidly increases and then gradually decreases. The dimensionless formula for calculating the flow velocity near the wall is obtained. The relationship between the arc fan flow geometric characteristics and the critical structural sizes of the arc fan nozzle is analysed.
3. When the water supply is constant, the average dedusting efficiencies for total dust and respirable dust reached 88.1% and 86.5%, respectively. Compared to the full cone nozzles previously used, the average removal efficiency for total dust increases by 34%, and the average removal efficiency for respirable dust increases by 32%. The dust removal efficiency is obviously improved.

## Abbreviations

CCD: Charge Coupled Device
*cl*_*g*_: The chord length of the guiding object
*cl*_*no*_: The chord length of the nozzle orifice
*H*_*ch*_: The maximum perpendicular distance from the vertex of the arc to *L*_*ch*_
*h*_*g*_: The height of the guiding object
*h*_*no*_: The height of the nozzle orifice
*L*_*ch*_: The maximum chord length of the cross section
*l*_*g*_: The length of the guiding object
RDC: Respirable dust concentrations
TDC: Total dust concentrations
VOF: Volume of fluid
